# Creating Enforceable Biosecurity Standards for Nucleic Acid Providers

**DOI:** 10.1049/enb2.70003

**Published:** 2025-12-17

**Authors:** Jacob Beal, Tessa Alexanian

**Affiliations:** ^1^ RTX BBN Technologies Cambridge Massachusetts USA; ^2^ International Biosecurity and Biosafety Initiative for Science (IBBIS) Geneva Switzerland

**Keywords:** biosecurity, bio‐economy, standardisation

## Abstract

Although there is broad agreement among biosecurity experts that nucleic acid synthesis providers should screen orders for potential threats, there is no agreed upon mechanism to verify whether a provider is implementing effective screening practices. This leads to economic costs for responsible actors, unverifiable claims of screening, and regulatory hesitation. The sequence biosecurity risk consortium (SBRC) aims to address these problems by developing a standard definition of ‘sequences of concern’ based on a scientific assessment of biosecurity risk from synthetic nucleic acids. Building on this effort, we outline a roadmap for moving from aspirational to enforceable standards via industry‐led standards development, with support and participation from government regulators.

## Graphical abstract

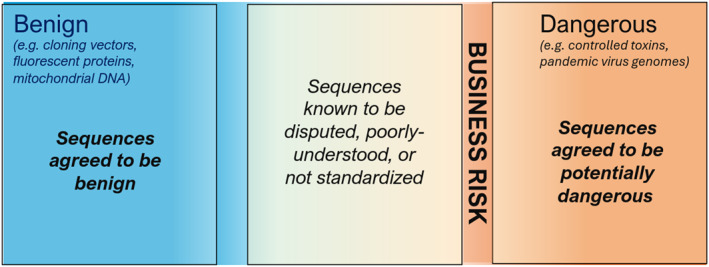

## 1 Introduction

There is broad agreement among biosecurity experts that nucleic acid synthesis providers should screen orders before sending out DNA or RNA that could facilitate the construction of dangerous biological agents [1, 2, 3]. However, there is no agreed upon mechanism to verify whether a provider is implementing effective screening practices. This leads to:

1. *Economic costs for responsible actors*. Providers must invest resources to develop and operate a screening system, and may find it challenging to remain cost‐competitive with providers that do not screen, especially given the risk of losing customers who prefer faster turnarounds and minimal review.
2. *Unverifiable claims of screening*. Even strong incentives for screening, which can level the playing field for responsible actors, must be supported by enforcement of minimum biosecurity standards. Existing frameworks based on voluntary guidance (as in the UK) and self‐attestation (as in the USA) have encouraged adoption of screening, but practices remain highly uneven.
3. *Regulatory hesitation*. Government agencies are reluctant to enforce screening in the absence of an agreed standard due to fears about stifling innovation, regulatory capture, and international fragmentation.

This perspective outlines a path to move from aspirational to enforceable standards via industry‐led standards development, with support and participation from government regulators.

We review key challenges in biosecurity for nucleic acid providers. We then present an approach to developing technical consensus on screening decisions and discuss the relationship between stages of adoption and policy change. Finally, we provide a roadmap for a structured public/private collaboration leading to the implementation of effective and enforceable biosecurity for nucleic acid providers in the United States and discuss recommended actions to implement this roadmap.

## 2 Synthesis Screening Can Create Conflict Between Biosecurity and Business

Industry leaders have recognised for nearly 20 years that some sequences, such as those that can reconstruct pathogen genomes, should only be sent to trusted customers. Many synthesis providers, including those following the International Gene Synthesis Consortium (IGSC) Harmonised Screening Protocol [4], screen their orders to recognise potentially risky sequences (sequence screening) and screening customers' identity and intended use for orders (customer screening). Synthesis screening has long been necessary to comply with export control regulations in over 50 countries, and is now recommended by national‐level guidance in multiple countries [5, 6], leading AI developers [7], an ISO standard [8], and the WHO [1].

Synthesis providers are thus currently expected to be responsible for detecting suspicious orders, which they should deny and, when appropriate, report to law enforcement or designated government agencies. Government, by contrast, is responsible for investigating whether there are bad actors behind suspicious orders and for determining what regulatory requirements are imposed on synthesis providers. At present, the primary enforcement mechanism in most jurisdictions is export control laws, in which providers are penalised only if government regulators determine that they have been shipping dangerous nucleic acids to foreign entities without appropriate licences.

So far, government regulators have primarily chosen to encourage adoption of synthesis screening with voluntary guidance. However, in 2024, the USA was the first country to require providers to self‐attest to compliance with screening practices [6], and in May 2025, the US White House issued an Executive Order on ‘Improving the Safety and Security of Biological Research’ [9] (EO 14292) ordering the 2024 ‘Framework for Nucleic Acid Synthesis Screening’ to be revised to:

[E]nsure it takes a commonsense approach and effectively encourages providers of synthetic nucleic acid sequences to implement comprehensive, scalable, and verifiable synthetic nucleic acid procurement screening mechanisms to minimise the risk of misuse. … To ensure compliance, the updated Framework shall incorporate the enforcement mechanisms described [elsewhere in the Order].

In July 2025, the US also recommended the following policy action to ‘Invest in Biosecurity’ as part of America's AI Action Plan:

Require all institutions receiving Federal funding for scientific research to use nucleic acid synthesis tools and synthesis providers that have robust nucleic acid sequence screening and customer verification procedures. Create enforcement mechanisms for this requirement rather than relying on voluntary attestation.

We believe the move towards verifiable claims of screening is positive. Without enforceable standards, screening becomes essentially voluntary, as claims of screening cannot be verified. Governments are also reluctant to enforce screening in the absence of an agreed standard due to fears about stifling innovation, regulatory capture, and international fragmentation. We wish to particularly highlight the conflict between biosecurity and business risk created if a regulatory regime requires providers to screen orders without clear guidance on which orders are acceptable to fulfil.

**FIGURE 1:**
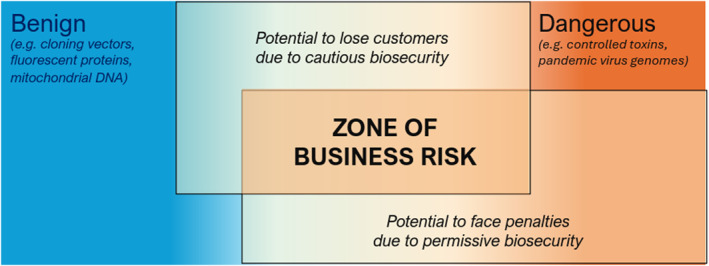
Without clear guidance, providers must risk losing legitimate customers who disagree with cautious risk assessments, or risk regulatory penalties if governments disagree with permissive risk assessments. Providers are thus incentivised to oppose strong regulatory enforcement regimes in the absence of a standard for decision guidance.

Although extreme cases are straightforward (e.g., complete toxin genes or large pathogen genome segments clearly warrant scrutiny, whereas ribosomal RNA clearly does not), many sequences fall into a zone where risk assessment is subjective and contentious (Figure 1).

The challenges of applying taxonomic lists of pathogens and toxins to sequence‐level risk are well‐documented [10], and include:

- Should sequences that are unique to a regulated pathogen, but which have found common benign uses in synthetic biology (such as IRES and 2A sequences) be flagged?
- Should sequences from non‐regulated organisms with strong homology to regulated toxins (such as heat‐labile *E. coli* enterotoxins with significant homology to Cholera toxin) be flagged?
- Should sequences from regulated organisms be flagged if those sequences are unique, but not known to be virulence factors? [11].

This subjective area is where it is most difficult for synthesis providers to make decisions about whether to fulfil orders. A provider taking a more cautious stance on the questions above risks losing legitimate customers who disagree with their risk assessments. At the same time, even providers with effective screening systems have no protection against fines or other regulatory penalties if government authorities later disagree with their risk assessments. In the status quo, synthesis providers are thus left to make independent judgement calls, whereas it is difficult for governments to enforce nucleic acid regulations without causing significant economic disruption.

The seemingly obvious way out would be for regulators to provide decision guidance at the granularity of specific sequences of concern, which is the level on which providers must evaluate orders. As early as 2006, the US National Science Advisory Board for Biosecurity (NSABB) recommended that the US government ‘provide clarification of what genetic elements or genomes are covered by [export controls]’ [12]. Industry organisations such as the IGSC have advocated for governments to establish sequence‐level guidance, with the leadership of Twist Bioscience calling, in 2021, for ‘a committed, ongoing effort to catalogue, in detail, the ways in which proteins and genetic networks can be used to cause harm in contexts subject to regulatory control… a database of risk‐associated sequences, their known mechanisms of pathogenicity and the biological contexts in which these mechanisms can cause harm’ [3].

Two decades of advocacy have not yet produced the institutional capacity to build and maintain such a database within any government, and we know of no current effort ongoing to build one in at least the USA, UK, or EU. At present, the UK screening guidance calls for ‘academics, industry, think tanks, and consortia’ to ‘develop a database for screening SOCs’ and ‘determine which sequences from pathogens should not cause concern and therefore do not need to be screened against’ [13]. In France, a 2023 update to the national regulation of hazardous microorganisms and toxins (MOT) classified specific bacterial genes as requiring licencing [14], but does not amount to a systematic database. In the USA, the Bureau of Industry and Security has responded to Commodity Classification Requests to determine whether specific sequences fall under export controls, and the National Institutes of Standards and Technology has worked with nucleic acid providers and screening tool developers to create a small blinded test set of sequences to facilitate inter‐tool screening comparison [15], but there remains a large zone of ambiguity in which biosecurity and business risk are in conflict.

We thus propose resolving the enforcement dilemma by organising industry‐led development of decision guidance that both policymakers and providers can embrace as a standard for enforcement.

## 3 Industry‐Led Development of Enforceable Sequence Standards

For decision guidance to function as an enforceable standard, which regulators and providers alike support, it must meet three essential criteria:

1. Synthesis providers must be confident that compliance with the guidance provides protection from enforcement penalties.
2. Governments must be satisfied that the standard is sufficiently comprehensive to fulfil their mandate of safeguarding public health and national security.
3. Guidance must be able to evolve as biological knowledge expands and the threat landscape shifts.

Critically, none of these require the problem of assessing biosecurity risk to be fully solved. It is sufficient for all of the key stakeholders to agree on ‘good enough’ ranges where enforcement can be expected and where it is safe to do business.

**FIGURE 2:**
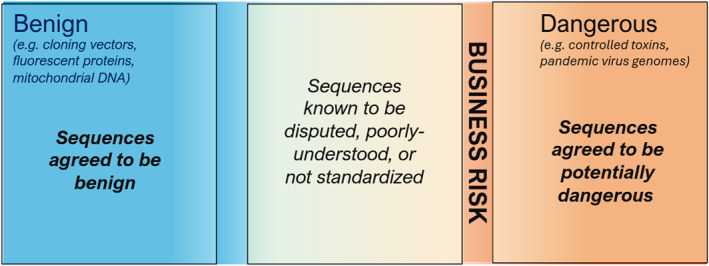
Both business and biosecurity risks can be reduced with a standard that makes the sequences explicit where there is no agreement currently.

To create such a standard, a group of nucleic acid providers, screening tool developers, policymakers, and scientific experts have created the Sequence Biosecurity Risk Consortium (SBRC) [16]. This consortium, which is currently being led by the authors and expands the prior IGSC working group nucleic acid screening test sets [17, 18], maintains a standard definition of ‘sequences of concern’ that explicitly demarcates areas of uncertainty. Specifically, the SBRC is developing a standard that categorises nucleic acid sequences into three general categories (Figure 2) adapted from prior work on cross‐tool comparison by the IGSC working group [18]:

- *No Flag*: Sequences agreed to be benign,
- *Flag*: Sequences agreed to be potentially dangerous, and
- *Disputed*: Sequences where there is known to be a lack of agreement.

Identifying sequences known to be disputed, poorly‐understood or otherwise outside agreement is critical to making this process work. That grey area in between contains materials that a government may not be comfortable declaring benign, but where the actual risks are not expected to be particularly high. Likewise, due diligence can be established for a biosecurity system based on its handling of benign and potentially dangerous sequences, and identifying the grey area reduces liability by acknowledging that even responsible actors may disagree on biosecurity assessment of such sequences. This makes the standard enforceable, because responsible actors can count on a lack of ‘trick questions’ from the identified area of disagreements, and can readily mitigate regulatory risk through their own due diligence.

Explicitly identifying disputed sequences also facilitates faster agreement on standard guidance. The IGSC working group on Nucleic Acid Screening Test Sets [18] established that current areas of agreement on biosecurity risk are quite substantial, with 76.4% of sequences agreed to be either benign or potentially dangerous [19]. The working group disagreed about 5.1% of the sequences (i.e., some members felt they should be flagged, whereas others felt they should not be flagged) and classified the remainder of the sequences as too poorly studied to be firmly categorised as benign or dangerous. This shows that biosecurity‐related risk can be mitigated for the majority of sequences that are not disputed, without having to wait to resolve disagreements about the small remaining fraction.

Finally, including a disputed category facilitates improvement of the standard over time. As disagreements are resolved or new knowledge is obtained, sequences currently lacking categorisation can be moved from the grey area into the benign or potentially dangerous categories. The category also allows a period of adjustment when sequences are recategorised (e.g., if a gene previously believed to be benign is found to participate in a newly‐characterised toxin synthesis pathway), as marking them as disputed allows a period of adjustment where their handling can be changed before they become part of due diligence requirements again under their new category.

We propose that the development of enforceable standards be industry‐led for several reasons. First, as noted in the previous section, governments have not built the institutional capacity to systematically characterise sequence‐level biosecurity risks. Second, government regulation of synthesis screening has typically fallen under the purview of security‐focused policymakers for whom it is easier to define conservative standards (categorising sequences as potentially dangerous) than to definitively permit some sequences as low risk. By contrast, industry has more incentive to propose benign sequences in order to reduce their compliance burden, and so industry‐led standards development will expand access to a ‘safe harbour’ of sequences where there is significant benefit to having them readily acquired by scientists conducting legitimate research, although still being subject to review by security‐focused policymakers.

Most critically, the synthesis industry is international, and yet synthesis screening regulation has thus far been, and will likely continue be, implemented on a country‐by‐country basis. If early‐adoption jurisdictions enforce overly cautious standards that are difficult for industry to implement, this could stifle innovation among responsible industry actors and discourage other jurisdictions from adopting standards. Additionally, stringent standards may be difficult for customers to accept, potentially leading to regulatory arbitrage and international ‘provider shopping.’ Thus, although governments must necessarily take the lead in some aspects of biosecurity, industry‐led standards also have a part to play in international coordination.

Establishing standard categorisations of nucleic acid sequences is well underway, and we believe that the definitions being produced by the SBRC can form the basis for testing of the full order screening process. However, as discussed below under “Roadmap and Dependencies”, enforceable biosecurity standards will require decision guidance related to customer legitimacy as well as sequence risk.

## 4 Stages of Adoption & Policy Change

**FIGURE 3:**
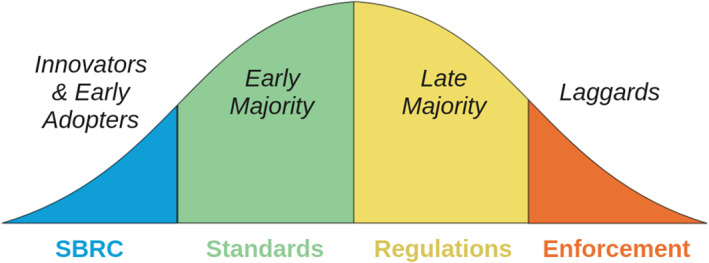
Adoption curve for synthesis screening standards. In the early stages, adoption can be driven by industry‐only actions, such as the SBRC and deployment of voluntary standards. Later stages depend on governments defining and then enforcing regulations.

The recommendations in this document aim for “enforceable” standards because we believe that universal adoption of synthesis screening is required given the risk of ‘provider shopping’ by bad actors, and that universal adoption will only be achieved with the aid of government‐backed enforcement. However, we believe that standards can be adopted by many nucleic acid providers before any government begins to undertake enforcement, and that policy change can be facilitated by ensuring that the roadmap leading to enforcement aligns with the incentives of these earlier adopters (Figure 3).

*Innovators and early adopters* for screening standards are the two dozen organisations already participating in the SBRC, which include all the major screening tool developers and a half‐dozen synthesis companies. These organisations have already decided that they have sufficient incentives to adopt the standards as they are developed.

The early majority consists of organisations that will act if they see potential competitive value in adoption of biosecurity standards. The critical question for these organisations will be the cost/benefit tradeoff for adoption, which varies based on the specific circumstances of organisations and their business models. Accordingly, for this stage, critical properties will include the ease of adoption of biosecurity tools and standards and the minimisation of impact on customer experiences. Examples of organisations expected to be in the early majority are the 19 organisations listed as publicly attesting to compliance with the 2024 US Framework [20] and the active members of the IGSC [21], who have some overlap with the previous list.

This stage will be facilitated by deploying industrial certification processes, similar to UL or LEED certification, by which an organisation can be officially and publicly recognised for implementing biosecurity. Pilot projects to allow synthesis companies to test their sequence screening systems and demonstrate baseline capability are being conducted by IBBIS and SecureDNA using the test sets developed by NIST in partnership with the IGSC and SBRC in 2024 [15]. Later projects involving an auditing process, with proficiency testing against a more comprehensive standard that covers both sequence and customer screening, will be necessary for organisations to demonstrate full implementation of synthesis screening. The competitive value in adoption of biosecurity standards will increase as it becomes publicly known that early adopters have obtained certification, and advocates within early majority organisations will find it easier to secure the resources for their organisations to adopt screening standards and achieve certification.

Governments do not necessarily need to act at the early majority stage, though the risks associated misuse of biotechnology means that governments that feel biosecurity is a pressing concern are likely to choose to help accelerate adoption through actions such as stating that the standard can be used to comply with existing regulations (e.g., export controls, GMO or dual‐use licences, BWC implementation measures) or by issuing voluntary guidance that aligns with the standards. Once the early majority has voluntarily adopted standards, it de‐risks enforceable regulation from the government, and indeed the early adopters and early majority become incentivised to support enforceable regulation, as it now becomes a competitive advantage for them and a disadvantage for their competitors who have been neglecting biosecurity.

For the late majority, credible and enforceable regulations being put in place should be sufficient, since their main barrier to standards adoption is typically not willingness but prioritisation. To convince the laggards to either adopt standards or exit the market, however, the government will need to take action against some non‐compliant companies (e.g., fines, cancelation of licences), showing that regulations are not just enforceable, but that there is a substantial business risk from enforcement action. Enforcement of regulations is also the mechanism by which bad actors (either negligent or intentionally permissive) amongst the synthesis providers can be detected and dealt with.

## 5 Roadmap for Establishing Enforceable US Standards

This manuscript was initially prepared in response to the May 2025 US Executive Order on ‘Improving the Safety and Security of Biological Research’ [9] (EO 14292) and the USA currently appears to be the closest jurisdiction to implementing enforceable standards with specific compliance mechanisms. The SBRC is an international consortium with members spanning multiple continents, and significant policy development is occurring outside the USA, including the UK's 2024 screening guidance [13] and ongoing discussion of synthesis screening within the EU [22]. The roadmap presented here could be adapted for other jurisdictions, and indeed international coordination will be necessary to prevent regulatory arbitrage and encourage harmonisation of standards for this global industry.

**FIGURE 4:**
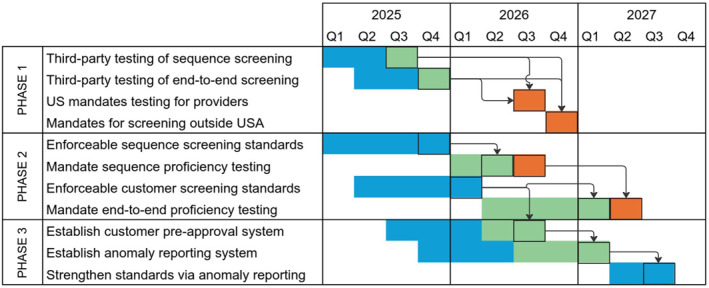
Multi‐phase roadmap and dependencies for establishing and improving enforceable screening standards. Actions are designated as primarily taken by industry‐led partnerships (blue), third‐party testing, certification and reporting organisations (green), and government regulators (orange).

Implementation of effective screening standards is time sensitive given the increasing biotechnological capabilities available to non‐state actors, particularly in light of recent AI developments. Accordingly, Figure 4 shows what we believe to be the fastest plausible roadmap for establishing and improving enforceable US screening standards. This roadmap was adjusted based on EO 14292, leveraging the order to mandate basic certification and testing, even before comprehensive standards will be ready to be used in certification processes.

The roadmap, based on our own analysis of the current state of development, the EBRC/NIST report on nucleic acid screening [23], and conversations with a diverse set of biosecurity stakeholders, shows three phases:

1. Verifying minimal compliance with screening requirements
2. Ensuring that screening systems are effective at biosecurity
3. Improving processes and standards to work at scale and address emerging threats

This roadmap calls for actions from industry‐led partnerships, empowered third‐party testing, certification, and reporting organisations, and government regulators. Industry‐led partnerships include actors such as the SBRC and IGSC, who are able to ensure that early standards reflect industry readiness and current best practices. The roadmap also calls for actions from independent third parties who are empowered (e.g., by standards agencies) to test and certify compliance with screening standards. Finally, only governments have the standing to actually enforce mandates. Implementation within the targeted timeline is highly feasible from a technical standpoint, but will depend strongly on supportive government actions, both directly in terms of actual enforcement and indirectly in terms of motivating resource investment from industry and independent third parties.

### 5.1 Phase 1: Verifying Minimal Compliance With Screening Requirements

In the first phase, EO 14292 is leveraged to require nucleic acid providers to undergo testing that checks that they have implemented at least rudimentary biosecurity screening capabilities. This begins with voluntary efforts to ensure both the synthesis industry and independent third parties are prepared for mandatory screening requirements:

1. *Voluntary third‐party testing of basic sequence screening* (Q3 2025): Basic proficiency at biosecurity flagging can be tested by independent third party organisations using the test sets developed by NIST in partnership with the IGSC and SBRC in 2024. [15] Test results should be renewed annually. The first such third party testing is, in fact, now available: as of October 2025, both IBBIS and SecureDNA have deployed portals where synthesis providers can undergo blinded testing and receive a pass/fail attestation. [24, 25].
2. *Voluntary third‐party testing of end‐to‐end screening* (Q4 2025): End‐to‐end screening is more difficult to test than sequence screening alone because conducting customer screening typically requires non‐automated engagement between humans, standards for decision‐making about customer legitimacy are less well‐established [26]. However, the EBRC is currently conducting end‐to‐end stress testing of nucleic acid synthesis providers [27], submitting orders to companies to learn about current sequence and customer screening practices. This could feed into the design of auditing processes by which independent third parties could certify that synthesis companies comply with requirements such as those in the IGSC Harmonised Screening Protocol [4] or ISO 20688‐2 [8]. Certification should be renewed every 1–3 years.

Once these voluntary efforts are complete, we believe that governments will be in a position to begin mandating that providers to go beyond self‐attestation in demonstrating compliance with screening requirements:

3. *US mandates certification of synthesis providers* (Q3 2026): Informed by the work on voluntary testing, the US Government should be able to establish a framework for accrediting third party organisations to certify that providers are in compliance with screening requirements and requiring that providers be certified.
4. *Mandates for certification or testing outside USA* (Q4 2026): We believe based on conversations with policymakers outside of the USA, including in the EU, UK, and New Zealand, that it is reasonable to expect mandates for certification to be established in at least some other countries during 2026 as well.

To be meaningfully enforceable, independent third parties will need to be designated by government regulators to conduct testing and certification. Given EO 14292, it is expected that the US Government will act most quickly in designating testing and certification organisations, but other governments such as the UK and EU may follow quickly once testing and certifications are being conducted, allowing them to endorse and align with an existing industry‐led framework rather than create their own separate framework. Finally, there will necessarily be some grace period provided in each jurisdiction between the time that testing and certification begins and the time when all nucleic acid providers are required to have complied.

### 5.2 Phase 2: Ensuring That Screening Systems Are Effective at Biosecurity

Because the accepted standards for flagging and customer screening are currently under‐defined, the testing and certification processes established in Phase 1 will not be adequate to ensure that nucleic acid providers actually have effective biosecurity processes. In Phase 2, testing and certification will be updated to use standards that ensure that all nucleic acid providers have ‘good enough’ biosecurity processes:

5. *Enforceable sequence screening standards* (Q3 2025): Proficiency testing can be strengthened to follow the SBRC ‘silver standard’, an updated and more robust definition of sequences of concern in the form of a ‘Biosecurity Flag Rubric’ and accompanying collection of test sequences, which has been completed as of September 2025.
6. *Mandate sequence screening proficiency testing* (Q2 2026): Once established, strengthened proficiency testing can be incorporated into testing for certification.
7. *Enforceable customer screening standards* (Q1 2026): The EBRC end‐to‐end stress testing project is expected to produce results that feed into a community process to agree on a ‘good enough’ standard decision guidance for customer screening. End‐to‐end screening processes can then be updated to require decision‐making to be at least as effective as the joint specifications for Biosecurity Flag Rubric sequence risk assessment and the standard decision guidance for customer screening.
8. *Mandate end‐to‐end proficiency testing* (Q4 2026): With the establishment of standard decision guidance for both sequence and customer screening, certification can begin to incorporate stress‐testing activities such as blinded submission of high‐risk orders through a provider's standard order interface.

At the end of Phase 2, we will have achieved a minimal implementation of effective and enforceable biosecurity standards. In addition, the SBRC is expected to continue to improve sequence of concern definitions throughout Phase 2 and beyond, including going ‘beyond the list’ to sequences of concern that are not covered by existing regulations. As these definitions continue to improve, proficiency testing should continue to incorporate them into testing requirements, following a short delay for implementation and deployment by providers.

### 5.3 Phase 3: Improving Processes to Work at Scale and Address Emerging Threats

In Phase 3, the goal is ongoing strengthening beyond ‘good enough’ towards a ‘gold standard’ and maintenance of efficacy in the face of ongoing evolution of the threat landscape. As noted above in the description of Phase 2, ongoing user‐funded activity of the SBRC will support continuous improvement of the Biosecurity Flag Rubric and strengthening of proficiency testing, including identification of threats ‘beyond the list.’ In this Phase, additional processes should also be introduced to improve scalability of screening and ensure it addresses emerging threats:

9. *Establish customer pre‐approval system* (Q3 2026): The standard decision guidance for customer screening can be used as a basis for establishing standards for registering customers as pre‐approved. This is highly desirable for customers that frequently order sequences of concern (e.g., vaccine developers and pathogen researchers), and for the nucleic acid providers that serve these customers. Development of the standard will be convened by organisations that are designated by governments to provide pre‐approval services. Once established, fees paid by customers wishing to be pre‐approved will sustain these services and also support the strengthening of customer screening standards.
10. *Establish anomaly reporting system* (Q1 2027): Once a customer pre‐approval system has been established, an anomaly reporting system can be put in place, similar to the Aviation Safety Information Analysis and Sharing (ASIAS) and the Medical Device Information and Analysis Sharing (MDIAS) systems. The reporting system will be operated by a third‐party organisation such as MITRE, which hosts the ASIAS and MDIAS systems, which will analyse anomaly reports for concerning patterns that can be reported to nucleic acid providers, as well as provide inputs for improvements in flagging and customer screening standards. This will likely require government funding to be sustained.
11. *Strengthen standards, including via anomaly reporting* (Q3 2027): As before, after an anomaly reporting system is in place, it can be incorporated into the requirements for certification, alongside continued improvement of proficiency testing.

## 6 Ongoing Efforts and Recommended Actions

The first stages of implementation of the recommended roadmap are already in progress. The SBRC is already in operation and made its first ‘enforcement ready’ standards release in September 2025, covering pandemic‐potential human viruses and regulated toxins [28]. The SBRC is also expanding the group of participating organisations, adding more nucleic acid providers, pathogen experts, regulators, and other stakeholders, as well as expanding to be more international in scope.

In a coordinated activity, NIST has been working with several organisations to pilot testing of sequence screening systems based on the test sets developed by NIST in 2024. A number of organisations, including NIST, EBRC, and IBBIS have also been working towards development of customer screening standards. Finally, SecureDNA has been experimenting with Exemption Certificates [29] that identify users who are allowed to have certain specific sequences and has launched discussions about how their approach might be generalised to a standard for supporting pre‐approval.

The SBRC has plans for sustaining its operations via user fees that, if realised, will enable the SBRC to implement its portions of the roadmap. Standards for proficiency testing and customer screening likewise appear to be plausible to implement without major new investments. The ambitious timeline for certification outlined above, however, would require a clear financial case for certifying organisations; although government formalisation of industry‐led standards may be sufficient, costs for early adopters could be reduced through subsidies to providers or if certifiers are given assistance to develop processes in advance of mandates. The customer pre‐approval and anomaly reporting systems in Phase 3, however, are expected to need additional external resource support to appropriate parties in order to be implemented and sustained.

Finally, while we note that the roadmap above is US‐focused, stakeholders from other jurisdictions are actively contributing to SBRC standards development, and regulatory development is ongoing in the UK, the EU, and several other regions. The announcement of EO 14292, which spurred the writing of the original version of this document, increased the likelihood that the USA will be the first country to require enforceable standards for nucleic acid providers, but another jurisdiction may get there first in the end. Going forward, it will be important to ensure that nucleic acid screening is implemented in all nations with significant nucleic acid synthesis providers, as well as ensuring that standards and regulations are well aligned internationally.

## Author Contributions

**Jacob Beal:** conceptualisation, methodology, writing – original draft, writing – review and editing. **Tessa Alexanian:** conceptualisation, methodology, writing – original draft, writing – review and editing.

## Funding

The study was supported by Sentinel Bio via support to the International Gene Synthesis Consortium (IGSC) and the International Biosecurity and Biosafety Initiative for Science (IBBIS).

## Conflicts of Interest

The authors are the current leadership of the Sequence Biosecurity Risk Consortium.

## Data Availability

Data sharing not applicable to this article as no datasets were generated or analysed during the current study.
